# Involvement of Organic Cation Transporter-3 and Plasma Membrane Monoamine Transporter in Serotonin Uptake in Human Brain Vascular Smooth Muscle Cells

**DOI:** 10.3389/fphar.2013.00014

**Published:** 2013-02-12

**Authors:** Rachel W. S. Li, Cui Yang, Y. W. Kwan, S. W. Chan, Simon M. Y. Lee, George P. H. Leung

**Affiliations:** ^1^Department of Pharmacology and Pharmacy, The University of Hong KongPokfulam, Hong Kong; ^2^School of Biomedical Sciences, The Chinese University of Hong KongShatin, Hong Kong; ^3^State Key Laboratory of Chinese Medicine and Molecular Pharmacology, Department of Applied Biology and Chemical Technology, The Hong Kong Polytechnic UniversityHung Hom, Hong Kong; ^4^Institute of Chinese Medical Science, The University of MacauMacau, China

**Keywords:** serotonin, organic cation transporter, monoamine transporter, vascular smooth muscle cells

## Abstract

The serotonin (5-HT) uptake system is supposed to play a crucial part in vascular functions by “fine-tuning” the local concentration of 5-HT in the vicinity of 5-HT_2_ receptors in vascular smooth muscle cells. In this study, the mechanism of 5-HT uptake in human brain vascular smooth muscle cells (HBVSMCs) was investigated. [^3^H]5-HT uptake in HBVSMCs was Na^+^-independent. Kinetic analyses of [^3^H]5-HT uptake in HBVSMCs revealed a *K*_m_ of 50.36 ± 10.2 mM and a *V*_max_ of 1033.61 ± 98.86 pmol/mg protein/min. The specific serotonin re-uptake transporter (SERT) inhibitor citalopram, the specific norepinephrine transporter (NET) inhibitor desipramine, and the dopamine transporter (DAT) inhibitor GBR12935 inhibited 5-HT uptake in HBVSMCs with IC_50_ values of 97.03 ± 40.10, 10.49 ± 5.98, and 2.80 ± 1.04 μM, respectively. These IC_50_ values were 100-fold higher than data reported by other authors, suggesting that those inhibitors were not blocking their corresponding transporters. Reverse transcription-polymerase chain reaction results demonstrated the presence of mRNA for organic cation transporter (OCT)-3 and plasma membrane monoamine transporter (PMAT), but the absence of OCT-1, OCT-2, SERT, NET, and DAT. siRNA knockdown of OCT-3 and PMAT specifically attenuated 5-HT uptake in HBVSMCs. It is concluded that 5-HT uptake in HBVSMCs was mediated predominantly by a low-affinity and Na^+^-independent mechanism. The most probable candidates are OCT-3 and PMAT, but not the SERT.

## Introduction

Serotonin [5-hydroxytryptamine (5-HT)] is not only a neurotransmitter in the central nervous system and digestive tract, but also a potent vasoconstrictor. In the cardiovascular system, 5-HT is mainly stored in platelets, thereby maintaining a low level of free-circulating 5-HT. 5-HT is released during platelet aggregation. The released 5-HT feeds back on the platelets to amplify the aggregation process and causes the contraction of vascular smooth muscle cells through the stimulation of 5-HT_2_ receptors (Vanhoutte, 1990).

Several studies have suggested that 5-HT may be involved in vascular diseases such as hypertension. For instance, arterial contraction to 5-HT is profoundly enhanced in hypertension in animals and humans (Wyse, 1984; Dohi and Lüscher, 1991; Hutri-Kähönen et al., 1999). Also, an increased plasma level of 5-HT has been measured in various models of hypertension (Soares-da-Silva et al., 1995; Krygicz et al., 1996).

Arteries can take up 5-HT. This mechanism is crucial to the vascular functions of 5-HT because it “fine-tunes” the availability of 5-HT at its cognate receptors. The action of 5-HT is supposed to be terminated after it is removed from its site of action and is taken up into the cytosol of vascular cells, where 5-HT is eventually metabolized by monoamine oxidase-A (Ni et al., 2004). Interestingly, two research teams have reported that the uptake of 5-HT is a prerequisite for the mitogenic effect of 5-HT on pulmonary arterial smooth muscle (Lee et al., 1991; Eddahibi et al., 1999). Being a protonated molecule, 5-HT cannot diffuse across the lipid bilayer of the cell membrane under physiological conditions. Therefore, transporter-mediated mechanisms play a key part in 5-HT uptake. Similar to neurons, the smooth muscle cells of the aorta contain a specific serotonin re-uptake transporter (SERT; Ni et al., 2004). 5-HT uptake in the rat aorta is inhibited by the SERT inhibitors fluoxetine and fluvoxamine (Ni et al., 2004). In addition, a significant part of 5-HT is taken up by rat extracerebral arteries via a high-affinity transporter, which is also consistent to the characteristics of SERT (Amenta et al., 1985). Moreover, the contracting effect of 5-HT on the aorta can be potentiated by SERT inhibitors (Ni et al., 2004). The SERT may also contribute to the regulation of vascular contractility under pathophysiological conditions. For instance, the SERT in pulmonary vascular smooth muscle is up-regulated by hypoxia (Eddahibi et al., 1999; Wanstall et al., 2003) and pulmonary hypertension (Eddahibi et al., 2001). Besides, SERT activities in deoxycorticosterone acetate salt- and *N*_ω_-nitro-l-arginine-induced hypertensive rats are lower than those in normal rats (Ni et al., 2006).

Although 5-HT can be taken up by blood vessels, many problems have not been resolved. First, 5-HT-induced vasoconstriction in the main pulmonary artery and mesenteric artery cannot be enhanced by SERT inhibitors (Wanstall et al., 2003). Second, 5-HT uptake is reduced, but not abolished, in the carotid artery, mesenteric artery, and aorta of SERT-knockout rats compared with wild-type rats (Linder et al., 2008a). Although SERT protein is expressed in veins, it is not functional, since the 5-HT uptake in vein cannot be significantly inhibited by classic SERT inhibitors (Linder et al., 2008b). Therefore, a non-SERT-dependent mechanism may be present which may make contributions to 5-HT uptake in certain vascular beds. It is particularly of interest to study the 5-HT uptake mechanism in cerebral arteries since 5-HT is considered to be involved in the regulation of the cerebral circulation, and is implicated in the etiology of cerebrovascular diseases such as migraine, vasospasm due to subarachnoid hemorrhage, and stroke (Parsons, 1991). Studies have also shown that the 5-HT-induced contraction is enhanced in the *basilar* arteries of hypertensive rats (Nishimura, 1996). Therefore, we sought to characterize the 5-HT uptake mechanism in human brain vascular smooth muscle cells (HBVSMCs) in the present study.

## Materials and Methods

### Culture of HBVSMCs

Primary culture of HBVSMCs were obtained from ScienCell Research Laboratories (Carlsbad, CA, USA) and cultured in the medium supplied by the same company, at 37°C in an atmosphere of 95% air and 5% CO_2_.

### 5-HT uptake

Experiments were carried out in HEPES-buffered Ringer’s solution containing (in mM): 135 NaCl; 5 KCl; 3.33 NaH_2_PO_4_; 0.83 Na_2_HPO_4_; 1.0 CaCl_2_; 1.0 MgCl_2_; 5 HEPES; and 10 d-glucose (adjusted to pH 7.4 or other pH as specified in the figures). Experiments were also carried out in Na^+^-free buffer containing (in mM): 140 *N*-methyl-d-glucamine (NMDG); 5 HEPES; 5 KH_2_PO_4_; 1.0 CaCl_2_; 1.0 MgCl_2_; and 10 d-glucose (pH 7.4).

Confluent monolayers of cells in 24-well plates were washed thrice in HEPES-buffered Ringer solution. Cells were pretreated with the monoamine oxidase-A inhibitor clorgyline (10 μM) for 10 min and 300 μL of Ringer solution containing [^3^H]5-HT (1 μM, 2 μCi/mL) was then added to each well for 30 min. To determine the passive uptake of 5-HT, monolayers of cells were incubated in buffer containing [^3^H]5-HT in the presence of excess non-radiolabeled 5-HT (1 mM). Plates were then rapidly washed three times with ice-cold phosphate-buffered saline (PBS) containing (in mM): 137 NaCl, 2.68 KCl, 1.47 KH_2_PO_4_, and 8.1 Na_2_HPO_4_ (pH 7.4). Cells were solubilized in 0.5 mL of 5% (v/v) Triton X-100. The radioactivity was measured by a β-scintillation counter. Protein content was determined by spectrophotometric means using a commercial bicinchoninic acid assay (Pierce Biochemicals, Rockford, IL, USA).

### RNA isolation and reverse transcription-polymerase chain reaction

Total RNA was isolated from HBVSMCs using TRIzol reagent (Invitrogen, Carlsbad, CA, USA). Two micrograms of total RNA were used for first-strand cDNA synthesis using random hexamer primers and Superscript II RNase H^−^ Reverse Transcriptase (SuperScript Pre-amplification System, Invitrogen). The resulting first-strand cDNA was directly used for PCR amplification. The primers for amplifying various transporters have been used in other studies (Martel et al., 2003; Tahara et al., 2006; Zhang et al., 2006) and their sequences are listed in Table 1. PCR amplification was carried out using PCR SuperMix (Invitrogen) with the following parameters: denaturation at 94°C for 30 s, annealing at 55°C for 30 s, and extension at 68°C for 45 s. Thirty cycles were completed. This was followed by a final extension at 72°C for 10 min. PCR products were analyzed by 1% agarose gel electrophoresis and visualized by staining with ethidium bromide. To semi-quantify the PCR products of nucleoside transporters, the optical density values of protein bands were normalized to those of β-actin.

**Table 1:** **Oligonucleotide sequence of the primers used for RT-PCR**.

	Primers	Corresponding nucleoside	Size (bp)
OCT-1 (accession no.: NM_003057)	Sense 5′-CTGTGTAGACCCCCTGGCTA-3′	408–770	363
	Antisense 5′-GTGTAGCCAGCCATCCAGTT-3′	
OCT-2 (accession no.: NM_003058)	Sense 5′-CCTGGTATGTGCCAACTCCT-3′	590–923	334
	Antisense 5′-CACCAGGAGCCCAACTGTAT-3′	
OCT-3 (accession no.: NM_021977)	Sense 5′-GACCAAGGATTTGAGAAAGTTG-3′	2067–2485	419
	Antisense 5′-AGGGAATCTGTGGCTCTAGG-3′	
SERT (accession no.: NM_001045)	Sense 5′-CATCTGGAAAGGCGTCAAG-3′	1112–1430	319
	Antisense 5′-CGAAACGAAGCTCGTCATG-3′	
PMAT (accession no.: AY485959)	Sense 5′-ATGGGCTCCGTGGGGAGCCAG-3′	148–547	400
	Antisense 5′-TGTGCAGGGTCAGTCTCTCC-3′	
NET (accession no.: AK312793)	Sense 5′-CTTCTGGCGCGGATGAA-3′	89–483	395
	Antisense 5′-ATGGGCAGATTTTCCAAACG-3′	
DAT (accession no.: NM_001044)	Sense 5′-AAGAGCAAATGCTCCGTGGGA-3′	133–502	370
	Antisense 5′-CCCTGTTGAACTGGCCGAGG-3′	
β-actin (accession no.: NM_001101)	Sense 5′-GGCGTGATGGTGGGCATG-3′	197–436	240
	Antisense 5′-CTGGGTCATCTTCTCGCG-3′	

### Western blotting

Human brain vascular smooth muscle cells were grown to confluence on 10-cm Petri dishes. The cells were washed three times with ice-cold PBS, scraped in 2 mL of 5 mM sodium phosphate, pH 8, with protease inhibitor cocktail [Sigma, St. Louis, MO; 1:100 (v/v)]. Cells were sonicated briefly and centrifuged at 3,000 *g* for 10 min to remove nuclei and unbroken cells. The resulting supernatant was centrifuged at 30,000 *g* for 30 min to pellet the crude microsomal membranes, which was resuspended in 5 mM sodium phosphate. The crude membranes were then resolved on 9% (w/v) SDS-polyacrylamide gels and electrotransferred onto nitrocellulose membranes. After blocking with 5% (w/v) non-fat dry milk in PBS overnight at 4°C, nitrocellulose membranes were incubated with the anti-organic cation transporter (OCT)-3 or anti-plasma membrane monoamine transporter (PMAT) antibody [1:100 (v/v) dilution in blocking solution], at room temperature for 2 h. Nitrocellulose membranes were then washed extensively with 0.02% (v/v) Triton X-100 in PBS. After washing, the membranes were incubated with the horseradish-conjugated goat anti-rabbit secondary antibody [1:5000 (v/v) dilution in blocking solution] at room temperature for 2 h. Excess secondary antibody was again washed, and the bound secondary antibody was detected by enhanced chemiluminescence (Western Blot Chemiluminescence Reagent Plus; NEN Life Science Products, Boston, MA, USA). Protein expression of β-actin was similarly detected with the monoclonal mouse anti-actin antibody (Chemicon, Temecula, CA, USA). The molecular size of OCT-3, PMAT, and β-actin are 62, 58, and 42 kDa, respectively. Optical density values of OCT-3 and PMAT bands were normalized to those of β-actin.

### siRNA knockdown of OCT-3 and PMAT

Human brain vascular smooth muscle cells were transiently transfected with siRNA specific for OCT-3 and PMAT (Qiagen Incorporated, Valencia, CA, USA) for 10–12 h with RNAifect Transfection Reagent (Qiagen), according to manufacturer’s instructions. HBVSMCs were then further cultured for 24–48 h before used for mRNA determinations and 5-HT uptake study.

### Materials

[^3^H]5-HT was purchased from Moravek Biochemicals (Brea, CA, USA). All antibodies were purchased from Abcam (Cambridge, UK). Primers for PCR were bought from Invitrogen (CA, USA). Other chemicals were purchased from Sigma–Aldrich (St. Louis, MO, USA).

### Statistical analyses

Data are means ± SEM and were obtained from at least three independent experiments. Statistical analyses of the data were carried out using the Student’s *t*-test or ANOVA (one-way and two-way), if appropriate. *P* < 0.05 was considered significant.

## Results

### Time-course and kinetics of 5-HT uptake in HBVSMCs

**Figure 1:**
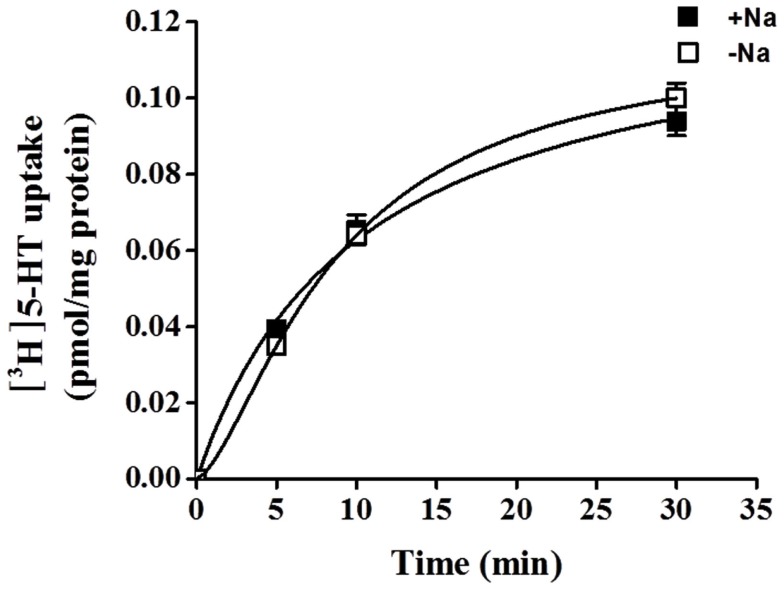
**Time-course of 5-HT uptake in HBVSMCs**. [^3^H]5-HT uptake (1 μM, 2 μCi/mL) was measured in HBVSMCs in the presence or absence of Na^+^ as indicated. Values are means ± SEM of three experiments carried out in triplicate.

**Figure 2:**
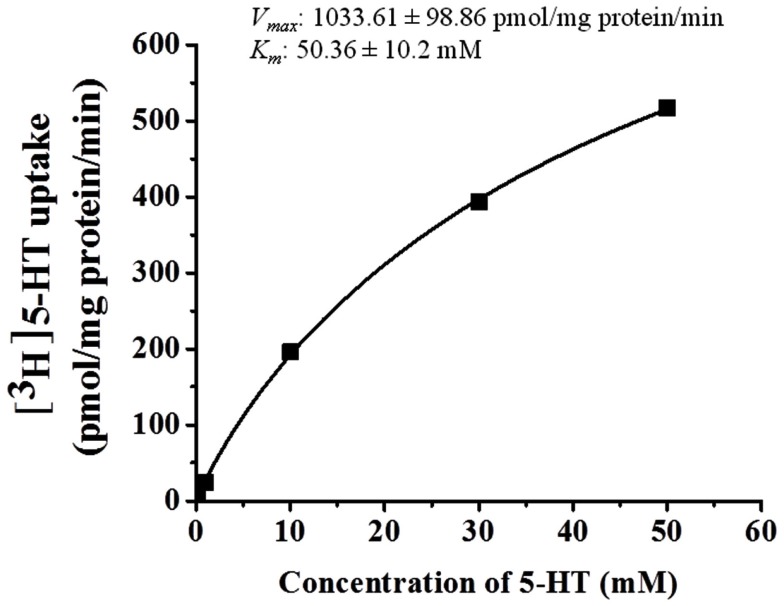
**Kinetic analyses of 5-HT uptake in HBVSMCs**. Concentration dependence of 5-HT (0.1 μM to 50 mM) uptake was determined by measuring [^3^H]5-HT uptake at room temperature for 30 min. Values are means ± SEM of three experiments carried out in triplicate.

5-HT uptake was measured in HBVSMCs after 0, 5, 10, and 30 min of incubation in [^3^H]5-HT (1 μM). Figure 1 shows that the uptake of 5-HT into HBVSMCs increased with time. There was no difference between Na^+^-dependent and Na^+^-independent uptake of [^3^H]5-HT. The kinetic parameters of 5-HT uptake were analyzed following a 30-min incubation in [^3^H]5-HT at various concentrations (0.1 μM to 50 mM). The rate of 5-HT uptake increased with increasing 5-HT concentration and yielded typical saturation kinetics (Figure 2). The estimated *K*_m_ of 5-HT uptake was 50.36 ± 10.2 mM and the estimated *V*_max_ was 1033.61 ± 98.86 pmol/mg protein/min.

### Effect of pharmacological inhibitors on 5-HT uptake in HBVSMCs

**Figure 3:**
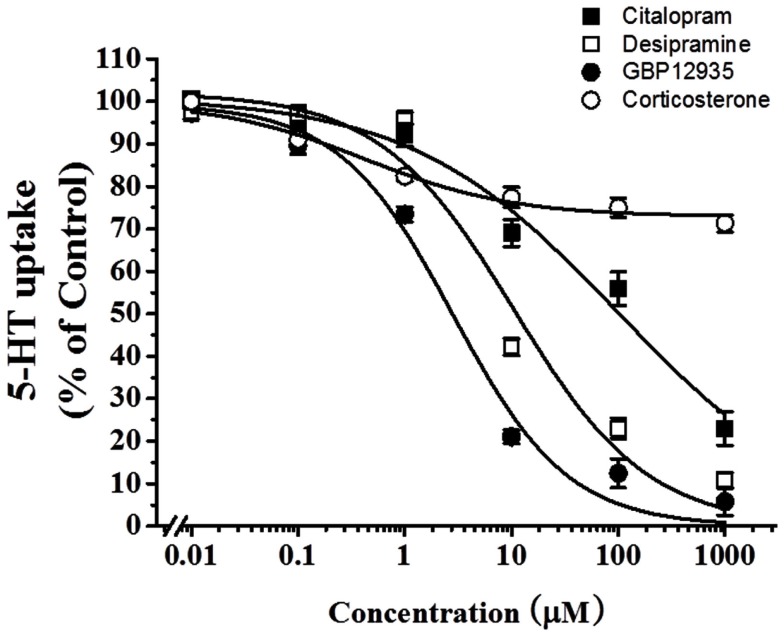
**Effects of various transporter inhibitors on 5-HT uptake in HBVSMCs**. [^3^H]5-HT uptake (1 μM, 2 μCi/mL) was measured at room temperature for 30 min in the presence of various concentrations of citalopram (■), desipramine (□), GBR12935 (●), and corticosterone (◯). Values are means ± SEM of three experiments carried out in triplicate.

To examine which type of transporters were responsible for 5-HT uptake in HBVSMCs, the effects of various inhibitors was studied. Citalopram (a specific SERT inhibitor), desipramine (a specific norepinephrine transporter (NET) inhibitor), and GBR12935 (a specific dopamine transporter (DAT) inhibitor) completely inhibited 5-HT uptake in HBVSMCs with IC_50_ values of 97.03 ± 40.10, 10.49 ± 5.98, and 2.80 ± 1.04 μM, respectively (Figure 3). The IC_50_ value for citalopram was significantly different from the that for GBR12935 (*P* < 0.05) but not for desipramine. The IC_50_ values for desipramine and GBP12935 were not statistically different. Corticosterone (a specific OCT-3 inhibitor) could only inhibited 5-HT uptake in HBVSMCs by 27%, with the threshold concentration between 10 and 100 nM.

### Identification of transporters in HBVSMCs by RT-PCR

**Figure 4:**
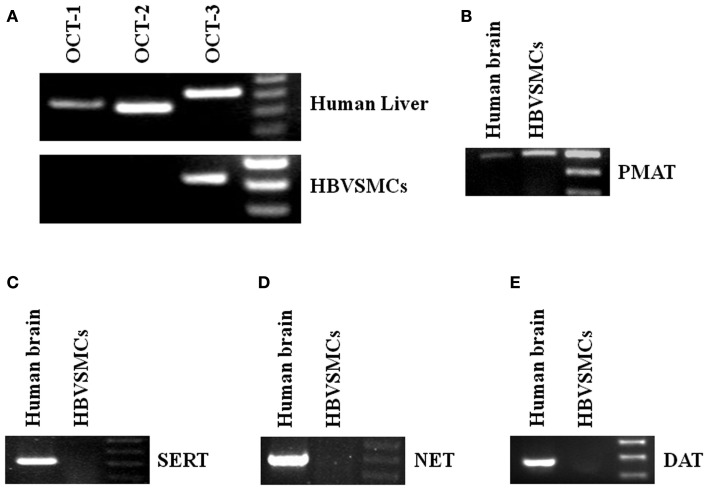
**Reverse transcription-polymerase chain reaction analyses of various 5-HT transporter mRNAs in HBVSMCs**. PCR products are seen in reactions using oligonucleotide primer pairs for **(A)** OCT-3 and **(B)** PMAT but not for **(A)** OCT-1, OCT-2, **(C)** SERT, **(D)** NET, and **(E)** DAT. Positive controls with human liver or brain cDNA indicate the expected sizes of amplified fragments: 363 bp (OCT-1), 334 bp (OCT-2), 419 bp (OCT-3), 319 bp (SERT), 400 bp (PMAT), 395 bp (NET), and 370 bp (DAT).

Reverse transcription-polymerase chain reaction (RT-PCR) was used to study the mRNA expressions of different transporters in HBVSMCs. cDNA from human livers and kidneys were used as positive controls because all the transporters studied were expressed in these tissues. The PCR products of OCT-3 and PMAT were amplified by RT-PCR from RNA isolated from HBVSMCs (Figure 4). The molecular sizes of PCR products were the same as expected and the PCR products were confirmed by sequencing, indicating the mRNA expressions of OCT-3 and PMAT in HBVSMCs. In contrast, the PCR product of OCT-1, OCT-2, SERT, NET, and DAT were not detected.

### siRNA knockdown of OCT-3 and PMAT

**Figure 5:**
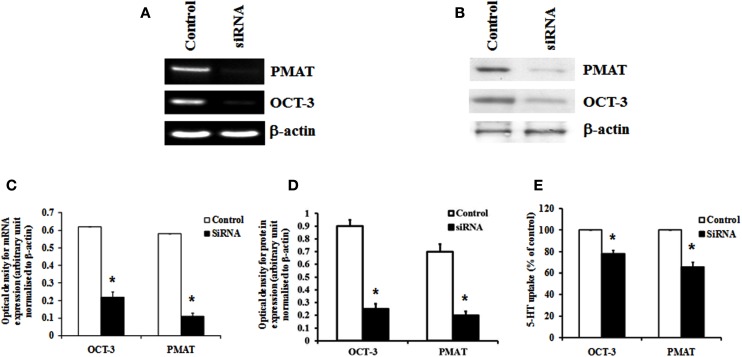
**Effects of siRNA knockdown of OCT-3 and PMAT on 5-HT uptake in HBVSMCs**. **(A)** mRNA and **(B)** protein expressions of OCT-3 and PMAT mRNA in HBVSMCs transfected with siRNA against OCT-3 and PMAT and a control non-silencing sequence. Bar graph showing the amount of **(C)** mRNA and **(D)** protein of OCT-3 and PMAT normalized to β-actin. **(E)** 5-HT uptake in HBVSMCs transfected with OCT-3 and PMAT siRNA and a control non-silencing sequence. Values are means ± SEM of three separate experiments. *P < 0.05 vs. control.

Organic cation transporter-3 and PMAT were knocked down specifically using siRNA to verify their contribution to 5-HT transport in HBVSMCs. Transfection of HBVSMCs with siRNA against OCT-3 resulted in a reduction in mRNA and protein expressions of 64.3 and 72.2%, respectively, as well as a reduction in 5-HT uptake of 22.1% in HBVSMCs. Transfection of HBVSMCs with siRNA against PMAT resulted in a reduction in mRNA and protein expressions of 81.2 and 71.5%, respectively, as well as a reduction in 5-HT uptake of 34.2% in HBVSMCs (Figure 5).

## Discussion

In the present study, we characterized the 5-HT uptake system in HBVSMCs (i.e., vascular smooth muscle cells cultured from human brain). Similar to the findings in certain vascular beds such as mesenteric arteries, vena cava, and jugular vein (Wanstall et al., 2003; Linder et al., 2008a,b), SERT does not have a significant contribution to 5-HT uptake in HBVSMCs. This conclusion can be supported by the fact that: (i) the mRNA of SERT is not expressed in HBVSMCs; (ii) 5-HT uptake in HBVSMCs is Na^+^-independent; (iii) the apparent affinity of HBVSMCs for 5-HT is much lower than that of SERT (*K*_m_, 1033 μM vs. 463 mM; Ramamoorthy et al., 1993); and (iv) citalopram at 100 nM (a dose that can inhibit SERT) does not affect 5-HT uptake in HBVSMCs. The lack of SERT activity in HBVSMCs could suggest that different mechanisms may be responsible for 5-HT uptake in different vascular beds.

It has been reported that 5-HT is one of the substrates of NET and DAT (Daws et al., 1998; Zhou et al., 2002). However, the present study has shown that the mRNA of NET and DAT are not expressed in HBVSMCs. BGR12935 and desipramine, which block DAT and NET in the nanomolar range, inhibited 5-HT uptake in HBVSMCs only at micromolar concentrations. Therefore, the involvement of NET and DAT in 5-HT uptake in HBVSMCs can be excluded. More likely candidates responsible for non-SERT-dependent 5-HT uptake in HBVSMCs are OCTs. OCTs are electrogenic and Na^+^-independent transporters (Koepsell et al., 2007). Substrates for OCTs can be as diverse as 5-HT, dopamine, norepinephrine, epinephrine, histamine, and cimetidine (Jonker and Schinkel, 2004). Three OCT isoforms, which share high sequence homologies and a common 12-transmembrane domain structure, have been identified and characterized from human and other mammalian species. They display considerable overlap in substrate specificity and are differentially expressed in various tissues. OCT-1 and OCT-2 are mainly expressed in the liver and kidney, respectively, where they are supposed to play a key part in systemic elimination of organic cations (Gorboulev et al., 1997; Zhang et al., 1997; Wright and Dantzler, 2004). OCT-3 (also called “extraneuronal monoamine transporter”) has a broad distribution and is found in the liver, heart, placenta, skeletal muscle, kidney, and brain (Gründemann et al., 1998; Wu et al., 2000). The results of RT-PCR in the present study revealed that the mRNA of OCT-3 (but not OCT-1 and OCT-2) is expressed in HBVSMCs. From a pharmacological perspective, OCT-3 can be distinguished from the SERT, DAT, NET, and other isoforms of OCTs by inhibition by corticosterone, *O*-methyl isoprenaline, and levamisole (Horvath et al., 2003; Martel and Azevedo, 2003). In the present study, corticosterone inhibited uptake of 5-HT by 27% and the threshold concentration was found between 10 and 100 nM, which is consistent to the characteristics of OCT-3 (IC_50_ = 0.29 μM; Hayer-Zillgen et al., 2002). Furthermore, siRNA knockdown of OCT-3 resulted in an inhibition of 5-HT uptake of 22% in HBVSMCs. All these data imply that OCT-3 may be involved in 5-HT uptake in HBVSMCs. However, the inability of corticosterone and siRNA against OCT-3 to abolish 5-HT uptake in HBVSMCs indicates the existence of other 5-HT transport systems in these cells.

Plasma membrane monoamine transporter shows homology to the equilibrative nucleoside transporter family but, unlike the existing equilibrative nucleoside transporter members that mainly transport nucleosides, PMAT specifically transports biogenic amines such as 5-HT and dopamine (Engel et al., 2004). The present study shows that the mRNA of PMAT is expressed in HBVSMCs. Also, the characteristic of 5-HT uptake activity in HBVSMCs, such as Na^+^ independence, low-affinity for 5-HT, and sensitivity to inhibitors of DAT, SERT, and NET in the micromolar range, coincide well with the properties of PMAT (Engel et al., 2004). Therefore, we cannot exclude the possibility that PMAT may also have a significant role in 5-HT uptake in HBVSMCs. A good pharmacological inhibitor for PMAT is currently unavailable. Although decynium-22 is a strong inhibitor of PMAT (*K*_i_ = 0.1 μM; Engel and Wang, 2005), it can inhibit OCT-3 with the same potency (Hayer-Zillgen et al., 2002). An alternative method to study PMAT is the use of siRNA. In the present study, 5-HT uptake was reduced by 34% after the siRNA knockdown of PMAT. This indicates that PMAT contributes to 5-HT uptake in HBVSMCs.

Other unidentified transporters or mechanisms independent of protein transporters may be involved in 5-HT uptake. 5-HT_2A_ receptors on the cell membrane can be internalized upon stimulation by 5-HT (Bhattacharyya et al., 2002; Gray et al., 2003). However, it is hitherto unclear if the internalization of 5-HT receptors is involved in 5-HT uptake, just like the well-known ability of the endothelin type B receptor to act as a clearance receptor for endothelin-1 (Fukuroda et al., 1994). Also, 5-HT is a very small molecule that can alternate its charge dependent upon pH. 5-HT may be able to cross the plasma membrane by passive diffusion. Nevertheless, based on the observation that pharmacological blockers and siRNA could greatly inhibit the 5-HT uptake in HBVSMCs, the involvement of transporter-independent mechanisms should not be significant.

The role of 5-HT in hypertension is controversial and intriguing. The enhancement of potency for 5-HT in inducing vasoconstriction or the pressor response have been observed in several models of hypertension, including DOCA-salt hypertensive rats (Watts, 1998), spontaneous hypertensive rats (Collis and Vanhoutte, 1981), and in human patients (Golino et al., 1991). Many mechanisms may contribute to this hyper-responsiveness, such as changes in 5-HT receptor signaling or changing circulating levels of 5-HT. It has been reported that the expression and function of 5-HT_2_ receptors are up-regulated in arterial smooth muscle in DOCA-salt hypertensive rats (Watts, 2002; Banes and Watts, 2003). In addition, SERT function in the aortas of DOCA-salt and LNNA-hypertensive rats is impaired (Ni et al., 2006). It may lead to an increase in the level of free-circulating 5-HT. However, the effect of the SERT on blood pressure is controversial because the systolic blood pressure, circadian rhythms in the heart rate and the gross level of motor activity of SERT-knockout rats are not different from those of normal rats (Homberg et al., 2006; Linder et al., 2008a). Such a discrepancy could be because 5-HT is taken up into arteries through mechanisms that are partially (but not completely) dependent upon the SERT, such as OCT-3 and PMAT as shown in the present study. The ability of OCT-3 to transport 5-HT has been demonstrated in SERT-deficient mice (Bengel et al., 1998; Schmitt et al., 2003). More importantly, animals may adapt to the genetic alteration. Activated or up-regulated mechanisms may serve the function of the ablated gene. It has been reported that OCT-3 contributes to 5-HT clearance if the expression of the SERT is low or absent. For instance, mRNA expression of OCT-3 is significantly increased in the intestine and hippocampus of SERT-deficient mice (Chen et al., 2001; Schmitt et al., 2003; Baganz et al., 2008). Therefore, OCT-3 may be important in the homeostatic regulation of extracellular 5-HT levels by ensuring (albeit low-affinity) uptake of 5-HT, particularly in the face of constitutively reduced expression or function of the SERT such as that seen in hypertension or during treatment with selective serotonin re-uptake inhibitors (Hirano et al., 2005; Mirza et al., 2007). The regulation of PMAT in SERT-knockout animals is not known. Investigating the possibility that chronic inactivation of the SERT also leads to compensatory up-regulation of PMAT will be interesting.

In conclusion, we demonstrated that 5-HT uptake in HBVSMCs is mediated predominantly by a low-affinity, Na^+^-independent, and pH-dependent mechanism. The most possible candidates are OCT-3 and PMAT. Unlike previous findings in the aorta, the SERT does not participate in 5-HT uptake in HBVSMCs. We hypothesize that OCT-3 and PMAT may have a role in regulating 5-HT functions by controlling the local concentration of 5-HT in the vicinity of 5-HT_2_ receptors in vascular smooth muscle cells. It is worth studying if impairment of OCT-3 and PMAT may be related to vascular diseases such as hypertension, ischemic stroke, arteriosclerosis, or even Alzheimer’s disease because this disease is not only a neurological but also a cerebrovascular disorder.

## Conflict of Interest Statement

The authors declare that the research was conducted in the absence of any commercial or financial relationships that could be construed as a potential conflict of interest.
